# Fingered Citron Volatile Oil: Extraction Methods, Chemical Composition, Biological Activity, and Product Development

**DOI:** 10.3390/cimb48060584

**Published:** 2026-06-01

**Authors:** Caiyun Sun, Shi Tang, Binlong Chen, Mo-Zi-Li Adu

**Affiliations:** 1 College of Animal Science, Xichang University, Xichang 615013, China; xcc20210212@xcc.edu.cn (C.S.); tangshi1989103@163.com (S.T.); 2 Research Center of Natural Resources of Chinese Medicinal Materials and Ethnic Medicine and Laboratory Service Center, Jiangxi University of Traditional Chinese Medicine, Nanchang 330004, China

**Keywords:** fingered citron volatile oil, *Citrus medica* L. var. *sarcodactylis*, extraction methods, chemical composition, biological activities

## Abstract

Fingered citron volatile oil is a volatile oil with a fresh and slightly floral aroma, extracted from fresh fingered citron. It is one of the natural perfume essential oils. At present, scholars at home and abroad mainly focus their research on fingered citron volatile oil on optimizing extraction processes, component analysis, pharmacological effects, and other aspects. With the continuous development and application of fingered citron volatile oil and related products, the research scope of fingered citron volatile oil is becoming increasingly extensive. As a narrative review, this paper summarizes and compares eight distinct extraction techniques for fingered citron volatile oil and clarifies the merits and drawbacks of each method. Steam distillation is one of the most employed approaches for volatile oil extraction in laboratory settings, whereas mechanical pressing is extensively applied and serves as the dominant and preferred industrial process for fingered citron volatile oil production. This paper also reviews its potential biological activities, including antidepressant, anti-anxiety, and sleep-improving effects, as well as the development and application of related products. Fingered citron volatile oil has been applied in products such as cigarettes and insecticides. Studying the chemical components of fingered citron volatile oil can not only help research its medicinal mechanisms but also promote the development of natural spices, providing a useful reference for the further utilization of fingered citron volatile oil in the future.

## 1. Introduction

Fingered citron (*Citrus medica* L. var. *sarcodactylis* Swingle), a variety of *Citrus medica* L. in the Rutaceae family, is the botanical source discussed in this review. This plant has several common names in Chinese literature and local usage, including names that have sometimes been translated as “bergamot” or “five-fingered citrus”. To avoid ambiguity with bergamot in English literature, which commonly refers to *Citrus bergamia*, this review uses “fingered citron” for the plant and “fingered citron volatile oil” for the corresponding volatile oil throughout the manuscript. When the fruit of fingered citron ripens, the carpels separate, forming uniquely shaped curved fruit segments that resemble human hands, hence its name [1]. The fruit of fingered citron has a golden peel and white and tender flesh, with a crisp, dry and sweet taste. Besides being eaten fresh, it can be made into preserves, candied fruits, and processed into fingered citron tea, fingered citron syrup, fingered citron jelly, and fingered citron fragrant wine. Its flowers, young fruits, and mature fruits can all be used as medicine, all of which have a rich fragrance. The peel of mature fingered citron is bright yellow, with a delicate and elegant fragrance that lasts long, and its fruit shape is unique, making it a precious flower for fragrance and fruit appreciation [2]. It has the effects of soothing the liver, regulating qi, harmonizing the stomach, and relieving pain, and is a valuable Chinese medicinal material [3,4].

In addition to the medicinal value of its fruit, fingered citron volatile oil, as a by-product, has also been studied by many scholars at home and abroad. Fingered citron volatile oil is present in the exocarp and part of the mesocarp of the fingered citron fruit, with a strong fresh fruit fragrance and a slightly sweet taste followed by bitterness [5]. The main extraction methods of fingered citron volatile oil include cold pressing, steam distillation, organic solvent extraction, and supercritical CO_2_ extraction, etc. The yields obtained by different extraction methods vary significantly, which can have a great impact on the quality of the essential oil. Steam distillation is widely used in the extraction of plant essential oils due to its simple equipment, low cost, and high output [6].

Volatile components account for approximately 93% to 96% of fingered citron volatile oil, mainly including monoterpenes, sesquiterpenes, and their oxygenated derivatives, such as limonene, γ-terpinene, α-pinene, β-pinene, β-myrcene, β-bisabolene, linalool, linalyl acetate, neral, geranial, neryl acetate, and geranyl acetate. Non-volatile substances account for approximately 4% to 7% and mainly include coumarins and furanocoumarins [7,8]. Previous studies have shown that the dominant constituents of fingered citron volatile oil are generally limonene and γ-terpinene, whereas linalool and linalyl acetate can be detected in some samples but should not be generalized as the major constituents of fingered citron volatile oil. The relative contents of these compounds vary substantially depending on cultivar, geographic origin, fruit maturity, harvest time, storage conditions, and extraction method [9,10].

Recent studies have shown that the pharmacological activities of many aromatic plants with medicinal value originate from the essential oil components in the plants, and the content of volatile essential oil in fingered citron reaches 16.0 mL/kg. On the one hand, fingered citron volatile oil has certain physiological activities and exhibits good efficacy in clinical practice. It can alleviate depression and anxiety, soothe emotions [10], and have effects such as relieving cough and asthma, and antioxidant activity [11]. On the other hand, with its fresh and fragrant aroma, fingered citron volatile oil, as a high-grade and precious natural spice, has good application value in industries such as food, cosmetics, and pharmaceuticals [12]. Nowadays, more experts and scholars are conducting research on fingered citron, but the research on the activity of fingered citron volatile oil is still not in-depth enough, and the development of fingered citron volatile oil is relatively insufficient. As a traditional Chinese medicinal plant, fingered citron still has great research and development space and value, and it has broad application prospects in the medical industry, health food industry, cosmetics, and daily necessities industry. This paper mainly summarizes and analyzes fingered citron volatile oil from four aspects: extraction methods of fingered citron volatile oil, chemical components, biological activities, and application prospects, aiming to lay a good foundation for the in-depth research on fingered citron deep processing and related fields.

## 2. Methods

This narrative review aimed to summarize the extraction methods, chemical composition, biological activities, and product development of fingered citron volatile oil. The literature search and selection process were designed to provide a broad and topic-focused overview rather than to conduct a systematic review or meta-analysis.

### 2.1. Literature Search Strategy

Relevant literature was searched in PubMed, Web of Science, ScienceDirect, SpringerLink, Wiley, ACS, Google Scholar, and China National Knowledge Infrastructure (CNKI). The main search terms included “fingered citron volatile oil”, “*Citrus medica* L. var. *sarcodactylis*”, “essential oil”, “volatile oil”, “chemical composition”, “extraction”, “biological activity”, “phototoxicity”, “safety”, and “product development”. Supplementary searches were performed using reported pharmacological activities and phytochemical components as keywords. Literature published or available before May 2026 was considered. No fixed lower publication-year limit was imposed; earlier studies were included when they provided foundational information on the traditional use, extraction methods, or chemical characterization of fingered citron volatile oil. This time frame was selected to include both foundational studies and recent publications relevant to this narrative review.

### 2.2. Literature Selection Criteria

For this narrative review, original experimental studies, phytochemical analyses, pharmacological studies, safety-related studies, product development studies, relevant reviews, Chinese doctoral and master’s theses, pharmacopeias, and classical Chinese medical texts related to fingered citron volatile oil were considered eligible. Literature was excluded if it was unrelated to fingered citron volatile oil, focused on unrelated Citrus species without clear relevance to fingered citron, lacked accessible bibliographic information or extractable information on extraction methods, chemical composition, biological activities, safety concerns, or product development, or represented duplicate publications.

### 2.3. Data Extraction and Presentation

Information on extraction methods, chemical constituents, biological activities, and product applications was extracted and narratively synthesized. Compound structures were redrawn using ChemDraw 19.0. The chemical classification and structural information of compounds were verified via the PubChem database (https://pubchem.ncbi.nlm.nih.gov (accessed on 15 March 2026)). Some schematic figures used graphical elements/icons from the BioGDP library (BioGDP.com). These elements were used only for schematic illustration and layout arrangement; the scientific content, figure organization, and final assembly were prepared by the authors. Adobe Illustrator 2026 was used for graphical refinement. No figures were directly reused or adapted from previously published articles.

## 3. Methods for Extraction and Isolation of Fingered Citron Volatile Oil

Currently, the main methods for extracting and separating fingered citron volatile oil include pressing, steam distillation (SD), molecular distillation (MD), solvent extraction (SE), supercritical fluid extraction (SFE), ultrasonic-assisted extraction, and simultaneous distillation-extraction (SDE), etc. The chemical components of fingered citron volatile oil are all heat-sensitive substances. In addition to containing a large number of terpenes that are prone to change, its main aromatic components, aldehydes, are also easily oxidized and deteriorated when heated. Therefore, the essential oil extraction technology must conform to the physical properties of fingered citron volatile oil. The research on extraction methods of fingered citron volatile oil focuses on developing methods with low cost, high efficiency and high oil yield. The component damage and low yield caused by traditional processes directly affect the product quality and economic benefits. Therefore, developing and researching new methods and continuously improving the existing ones are important approaches to expand the essential oil market. How to balance the component retention and extraction efficiency of fingered citron volatile oil has become a popular research hotspot in recent years (Table 1).

### 3.1. Cold Pressing Method

Pressing is an extraction method that squeezes oil from oil materials by means of mechanical external force. Since no heat is applied during the processing, it is also called cold pressing. The essential oil produced by cold pressing is a pale-yellow liquid with an oil yield of 1.0–1.6%. However, it has a better aroma, which is closer to the natural fresh citrus fragrance. The pressed residue can still be used to extract part of the citrus oil by steam distillation. Cold pressing is suitable for large-scale, continuous industrial production of citrus essential oils [21]. Common pressing methods include the sponge method, rasping and pressing method, and mechanical pressing method. Among them, the mechanical pressing method is a widely used pressing method in current production, and it is the main means and preferred process for extracting fingered citron volatile oil. Generally, cold pressing is carried out in an environment below 60 °C, which allows for the most complete retention of nutrients. The finished plant essential oils extracted by cold pressing technology are of high quality, maintaining their purely natural properties. Meanwhile, this method avoids the production of harmful substances that occur during high-temperature oil processing and retains the physiologically active substances in the oil as much as possible, thus meeting the “green” standards. However, cold pressing technology in China is still immature, and breakthroughs are needed in aspects such as the research and development of production equipment and the optimization of extraction processes [22].

### 3.2. Steam Distillation Method

At present, steam distillation (SD) is one of the most used methods for extracting volatile oils in laboratories. The principle of extracting volatile essential oils by steam distillation is an extraction method where medicinal materials containing volatile components are co-distilled with water, allowing the volatile components to be distilled out together with the water vapor, and then the volatile components are separated by condensation. This method is suitable for extracting chemical components that are volatile to a certain extent and have low solubility in water. The optimal process for extracting fingered citron volatile oil by steam distillation is as follows: do not add NaCl, use a solid–liquid ratio of 1:14, and set the distillation time to 7 h. This method has the advantages of a simple process, low cost, stable yield, no organic residue, good quality of volatile oil without impurities, etc. However, it also has drawbacks: the long-term high temperature during the distillation process may cause some substances in the volatile oil to be destroyed and easily hydrolyzed to generate useless impurities [13]. In addition, as an environmentally friendly solvent, water does not pollute the environment or essential oil products. The prepared fingered citron volatile oil has a pure flavor and can be directly applied in fields such as food. Currently, reducing energy consumption and improving extraction efficiency are the issues that need to be addressed in the application of this method. Some studies have used steam distillation to extract fingered citron volatile oil, with a yield of 9.7% from fingered citron. After GC-MS analysis of the components, it was found that the extracted essential oil contains about 15 components, among which limonene, linalool, and linalyl acetate have the highest contents [23].

### 3.3. Molecular Distillation Method

MD is a new type of liquid–liquid separation technology developed in recent decades, which is widely applied in fields such as food, medicine, and daily chemicals, especially in the extraction and separation of natural substances [14]. Different from traditional distillation, which relies on the principle of separation based on boiling point differences, molecular distillation operates by heating materials under high vacuum conditions and separating substances based on the differences in the mean free paths of molecules escaping from the liquid surface. Generally, lighter molecules have longer mean free paths and can easily reach the condensation surface, while heavier molecules have shorter mean free paths and collide with each other before reaching the condensation surface, returning to the solution. This process achieves the separation of light and heavy molecules [24]. Molecular distillation has the advantages of low distillation pressure, short heating time for materials, low operating temperature, high separation efficiency, good product quality, minimal change in components before and after distillation, and the ability to avoid organic solvents in the separated products. It is widely used in the separation and purification of high-boiling-point and heat-sensitive mixed materials [25]. Hu Anfu conducted a study on the separation and purification of fingered citron volatile oil using molecular distillation technology. Taking *α*-pinene (a characteristic aroma substance of fingered citron volatile oil) and limonene (the main component) as the target products, he analyzed the effects of distillation temperature, vacuum degree, scraper rotation speed, and feeding rate on the separation effect. Through orthogonal experiments, the optimal process conditions were obtained: distillation temperature of 35 °C, vacuum degree of 200 Pa, scraper rotation speed of 250 r/min, and feeding rate of 0.3 L/h. Under these conditions, the content of the target components increased from 44.2% to 75.3% [26].

### 3.4. Solvent Extraction Method

Solvent extraction, also known as solvent leaching, primarily utilizes the low boiling points of volatile components. Organic solvents are used to perform cold or hot extraction on plant materials, followed by evaporating the solvent through vacuum rotary evaporation to obtain crude essential oil. Appropriate organic solvents are selected based on the principle of “like dissolves like.” This method features simple equipment and high essential oil yields. However, it consumes large amounts of organic solvents, and substances such as resins, fats, and waxes in the plant are also extracted simultaneously, resulting in the essential oil containing relatively more impurities. Therefore, further refining is necessary. Gong Shengzhao et al. [27] conducted a study on extracting essential oil from Shatian pomelo peels and obtained the optimal process conditions for extracting pomelo peel essential oil: using petroleum ether as the extraction solvent, a solid-to-liquid ratio of 24:1, reflux extraction twice, for 60 min each time. The essential oil yield was approximately 1.96%, with a relative density of 0.1857, an optical rotation of +97b, and a refractive index of 1.4765.

### 3.5. Supercritical Fluid Extraction Method

In recent years, SFE, as a new type of separation technology, has been widely applied in the processing of natural products. It is an extraction method that uses supercritical fluid as the extractant to separate the extract from the matrix, with carbon dioxide being the most used supercritical fluid. This method is suitable for fat-soluble, high-boiling-point, and heat-sensitive components, and is now widely used in the research of volatile components. The extraction of plant essential oils using supercritical extraction technology has advantages such as a simple process, few steps, high yield, environmental friendliness, and simple craftsmanship. However, it requires large equipment investment and high technical requirements for the process. Mira et al. [15] extracted citrus essential oil using the supercritical fluid method. GC-MS analysis showed that the extracted components mainly included oxygenated compounds, terpenes, limonene, and linalool. Supercritical extraction can achieve fractionation of essential oil components and reduce or eliminate terpene compounds that degrade essential oils, which is incomparable with traditional methods.

### 3.6. Ultrasonic-Assisted Extraction Method

Ultrasonic extraction refers to extraction with the assistance of ultrasonic waves and solvents. Through the high-speed, intense cavitation effect, mechanical vibration effect, and thermal effect generated by sound waves, it accelerates the diffusion process of target components from raw materials into the solvent, destroys the cells of plant materials, promotes the penetration of solvents into the material cells, shortens the extraction time, and improves the extraction rate. This method offers several advantages, including high extraction efficiency, short extraction time, simple operation, wide applicability, and low solvent consumption. However, it also has drawbacks such as high initial investment and equipment costs, limited component selectivity, and potential non-specific destruction of cell structures by the cavitation effect, which may lead to the simultaneous dissolution of impurities and target components, increasing the difficulty of subsequent separation and purification. Additionally, it generates certain noise pollution. Yan Zankai compared steam distillation with ultrasonic-assisted steam distillation. Under the same temperature and time conditions, the absorbance values of the test solutions extracted by ultrasonic-assisted steam distillation and steam distillation were 0.568 and 0.501, respectively, indicating that ultrasonic-assisted steam distillation has higher extraction efficiency [3].

### 3.7. Simultaneous Distillation-Extraction Method

SDE is a sample pretreatment technology that integrates distillation and extraction. Its core principle is to realize the efficient transfer of target components from samples to organic solvents through the synchronous operation of steam distillation and solvent extraction, and it is especially suitable for the analysis of trace volatile substances. Through the coupling of distillation and extraction, it has unique advantages in the field of separation and enrichment of volatile components and is especially suitable for scenarios that require simultaneous separation, concentration, and solvent conversion. Despite its disadvantages, such as long operation time and complex equipment, its irreplaceability in flavor analysis and natural product research makes it one of the commonly used technologies in laboratories. In practical applications, it is necessary to select an appropriate pretreatment method according to the properties of target components and the sample matrix. Zhao Xingjie extracted fingered citron volatile oil by using cold pressing, steam distillation, organic solvent reflux, and simultaneous distillation-extraction methods, and compared the yields of fingered citron volatile oil obtained by different extraction methods. The order was as follows: simultaneous distillation-extraction method > steam distillation method > organic solvent extraction method > cold pressing method [16].

### 3.8. Enzyme-Assisted Extraction

Enzyme-assisted extraction is a technology that utilizes the catalytic effect of enzymes to improve the extraction efficiency of target components. It is widely applied in fields such as natural product extraction. To enhance the extraction rate of fingered citron volatile oil, the use of cellulase [17] for assisted extraction is undoubtedly an effective method worth trying. Enzyme-assisted extraction is usually carried out under relatively mild conditions such as moderate temperature and pH values, which avoids the destruction of target components caused by high temperature, strong acid, strong alkali and other conditions in traditional extraction methods, and is conducive to maintaining the activity and stability of target components. Ouyang Hui et al. [18] conducted research on the extraction and process of essential oil from Xiangxi ponkan peel using enzyme-assisted extraction in 2010. Enzyme-assisted extraction also has advantages such as simple operation, environmental friendliness and non-toxicity, making its application in essential oil extraction more extensive [19].

## 4. Chemical Composition of Fingered Citron Volatile Oil

Fingered citron volatile oil is mainly composed of volatile components (93–96%) and non-volatile components (4–7%). The volatile components are primarily monoterpenes, sesquiterpenes, and their derivatives, while the non-volatile components are mostly coumarins and lactones [8,9]. At present, the analysis of the chemical components of fingered citron volatile oil mainly uses the GC-MS (gas chromatography–mass spectrometry, GC-MS) method. Marzocchi et al. [28] detected 42 compounds in the volatile components of fingered citron volatile oil by SPME-GC/MS. The monoterpene components include limonene, α/β-pinene, γ-terpinene, ocimene, linalool, and linalyl acetate; the sesquiterpene components include α-caryophyllene, β-caryophyllene, and β-bisabolene. Donoto et al. [29] determined the flavonoids in fingered citron volatile oil by using high-performance liquid chromatography-ion trap time-of-flight mass spectrometry (HPLC-IT-TOF-MS). The results showed that fingered citron volatile oil contains 4 polymethoxyflavones, namely sinensetin, 4′,5,6,7-tetramethoxyflavone, nobiletin, and tangeretin. They further determined the contents of nobiletin and tangeretin in cold-pressed fingered citron volatile oil. The main chemical components in fingered citron volatile oil are shown in Table 2.

## 5. Research on the Pharmacological Activities of Fingered Citron Volatile Oil

Volatile oil is a secondary metabolite fraction extracted from plants, also known as essential oil or aromatic oil. It is a complex mixture of volatile metabolites that contributes substantially to the characteristic aroma of aromatic plants. Fingered citron volatile oil is a metabolite mixture with multiple biological activities and potential application value [42]. Available studies suggest that fingered citron volatile oil and related citrus essential oils have potential biological activities, including effects on anxiety, depression, inflammation, oxidation, cough and asthma, insomnia, skin conditions such as vitiligo and acne, and other application areas [43].

### 5.1. Anti-Anxiety

Anxiety is a restless emotion that refers to a series of psychological, physiological, and behavioral reactions exhibited when the health or survival of an organism is threatened. It is a common response to psychological stress and includes components such as tension, restlessness, unease, and panic. Oxidative stress and inflammation are related factors contributing to anxiety [44]. The traditional treatment for anxiety disorders involves the use of benzodiazepines, but these drugs can cause side effects such as dizziness, drowsiness, and fatigue. In contrast, plant essential oils have few to no toxic side effects, are easily absorbed, and do not induce drug resistance, thus garnering increasing attention from researchers. Rombolà et al. [45] investigated bergamot essential oil from *Citrus bergamia* in rats using the open field test, elevated plus maze test, and forced swimming test. The results showed reduced locomotor activity and escape attempts and increased immobility, suggesting sedative and anxiolytic-like effects. Because this study was performed using *Citrus bergamia* essential oil, it is cited here as background evidence for related citrus essential oils, and direct validation using fingered citron volatile oil remains necessary.

### 5.2. Antidepressant

**Figure 1:**
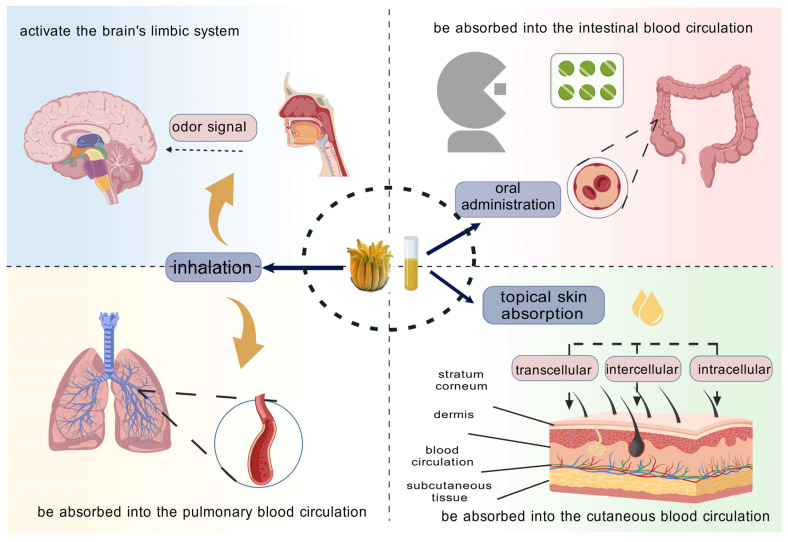
Routes of administration of fingered citron volatile oil in intervening depression. Created with BioGDP.com [50].

Depression is a common mental disorder, characterized by components such as low mood, slow thinking, reduced will to engage in activities, and feelings of inferiority. An imbalance of the two neurotransmitters of 5-hydroxytryptamine (5-HT) in the body is closely related to depression. With the continuous development of society, people’s life pressure is increasing, and the incidence rate of depression is also gradually rising. The search for natural antidepressant drugs with lower toxicity and higher efficacy has become a new research hotspot. Aromatic drugs can be used as a medium to improve traditional “aromatherapy”. Aromatic substances are made into inhalants, essential oils, etc., which enter the body through respiration or the skin to relieve mental stress, treat diseases and promote human health. Due to the special properties of plant essential oils, they can be used in various ways, such as smearing, rubbing, gargling, medicated baths, inhalation, etc., which usually involve multiple organs and tissues such as the nasal cavity, skin, lungs, and gastrointestinal tract. To achieve rapid efficacy, comprehensive consideration can be given to the physical and chemical properties, mechanisms of action, and administration routes of essential oils to give full play to the various advantages of plant essential oils [46]. The volatile oil extracted from fingered citron fruits has now become an important raw material for making essential oils, which can soothe the mood and relieve stress, and has potential medicinal value [47]. The common administration routes of fingered citron volatile oil for intervening in depression are shown in Figure 1.

**Figure 2:**
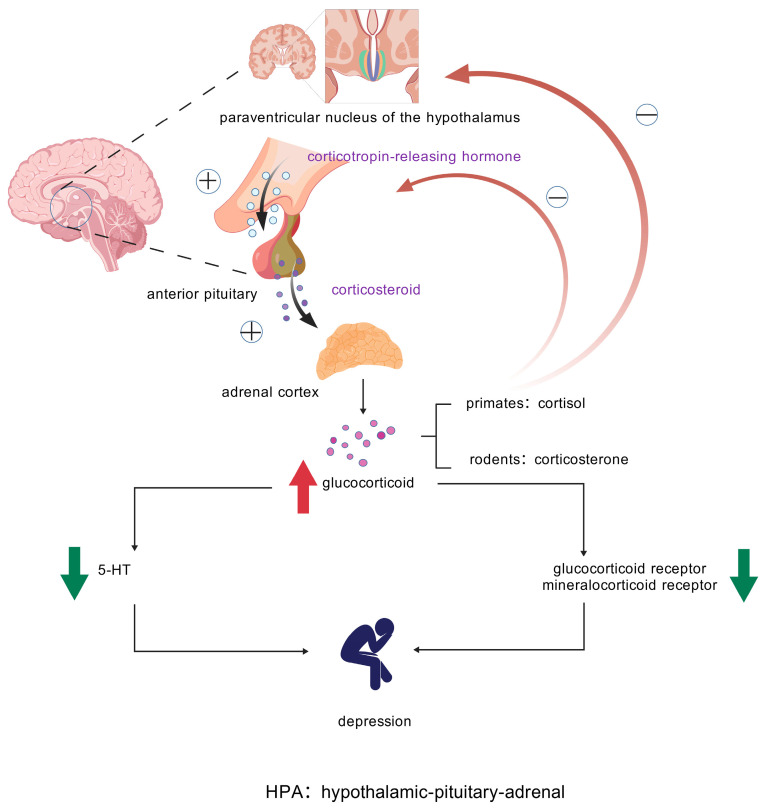
Related mechanisms between HPA axis and depression. The arrows in the figure are used to indicate the direction of the HPA-axis pathway and related regulatory effects. Specifically, black arrows indicate the direction of signal transmission or downstream effects; curved arrows indicate feedback regulation; the red upward arrow indicates an increase in glucocorticoid levels; and green downward arrows indicate decreases in 5-HT levels and glucocorticoid receptor/mineralocorticoid receptor expression or activity. Created with BioGDP.com [50].

Abnormality of the HPA axis is one of the important causes of depression [48]. Studies have shown [49] that research on the antidepressant effect of fingered citron volatile oil, based on the characteristics of fingered citron, found that fingered citron volatile oil contains a variety of components, mainly limonene, pinene, terpinene, etc. Among them, limonene is the main aroma component that exerts the antidepressant effect. The antidepressant mechanism of fingered citron volatile oil remains to be further studied, and its mechanism may be related to reducing the excitability of the HPA axis (Figure 2).

### 5.3. Antidepressant

Insomnia has become a prevalent and serious health issue today. As another essential oil proven to be effective for insomnia besides lavender essential oil, fingered citron volatile oil has attracted increasing attention for its unique role in promoting sleep. It has been reported that fingered citron volatile oil can promote sleep by activating the parasympathetic nervous system [51]. In view of the psychological pressure brought by the COVID-19 pandemic and its adverse impact on individuals’ sleep quality, Nobuyuki et al. [52] invited 48 college students to participate in a prospective randomized controlled trial, aiming to explore whether fingered citron volatile oil can alleviate COVID-19-induced psychological pressure, improve sleep quality and reduce the number of morning awakenings in the short term. The volunteers inhaled fingered citron volatile oil or placebo spray before going to bed and after waking up respectively. The results of the sleep scale and stress scale showed that, compared with the placebo group, the volunteers using fingered citron volatile oil performed significantly better in multiple sleep quality indicators such as “reduced drowsiness upon waking”, “feeling more refreshed upon waking” and “extended sleep duration”. Therefore, fingered citron volatile oil is regarded as a natural therapy that can effectively promote physical and mental relaxation and improve sleep quality. In the prevention and treatment of insomnia, essential oil aromatherapy is highly praised for its advantages of simplicity, convenience, safety, and effectiveness. Although essential oils have shown significant efficacy in improving sleep, their specific mechanisms lack in-depth research and still require a large amount of experimental data for verification [53].

### 5.4. Anti-Tumor

Cancer is the second leading cause of death worldwide. Currently, clinical treatments for cancer include surgery combined with radiotherapy and chemotherapy. Although these treatments have certain efficacy, the problem of multidrug resistance during chemotherapy seriously affects clinical outcomes [54]. Citrus extracts have anti-cancer activity. Bergamottin, as a non-volatile component in fingered citron volatile oil, has an inhibitory effect on malignant tumors such as lung cancer, intestinal cancer, breast cancer, and nasopharyngeal cancer [55,56,57]. The pathways by which fingered citron volatile oil inhibits cancer include inducing cancer cell cycle arrest and proliferation, inducing cancer cell autophagy, regulating protein signaling pathways to inhibit cancer cells, and inducing cancer cell apoptosis [58]. Fingered citron volatile oil has an inhibitory effect on MDA-MB-435 human breast cancer cells. Low and medium concentrations of the volatile oil can arrest MDA-MB-435 human breast cancer cells in the S and G1/M phases, causing cell apoptosis, while high concentrations of fingered citron volatile oil can lead to cell necrosis [59].

### 5.5. Antitussive, Antiasthmatic, and Expectorant Effects

Volatile oils have good antitussive and expectorant effects, and studies have also found that fingered citron volatile oil has a certain therapeutic effect on asthma. Terpinolene, β-myrcene, and phellandrene have significant anti-inflammatory, expectorant, and antitussive effects. Geraniol is clinically used to treat chronic tracheitis and can enhance the body’s immune function. Limonene is the main component of fingered citron volatile oil, and studies have found that it has significant antitussive and expectorant effects, indicating that limonene is one of the active components responsible for the antitussive and expectorant effects of fingered citron [10]. Shi Changchun et al. [60] extracted fingered citron volatile oil using steam distillation. Guinea pigs and mice in the experimental groups were pre-administered high, medium, and low doses of fingered citron volatile oil by gavage, while animals in the model control group were gavaged with 9 g·L^−1^ saline. In addition, acute branch syrup, aminophylline, and fresh bamboo juice were used as positive drug control groups for antitussive, antiasthmatic, and expectorant effects. The results showed that the cough latency periods in the high, medium, and low dose volatile oil groups and the acute branch syrup group were all longer than that in the model group. The asthma latency periods of guinea pigs in the high, medium, and low dose volatile oil groups induced by histamine inhalation were all longer than that in the model group. The tracheal phenol red excretion in the high, medium, and low dose volatile oil groups was significantly higher than that in the model group.

### 5.6. Anti-Inflammatory

**Figure 3:**
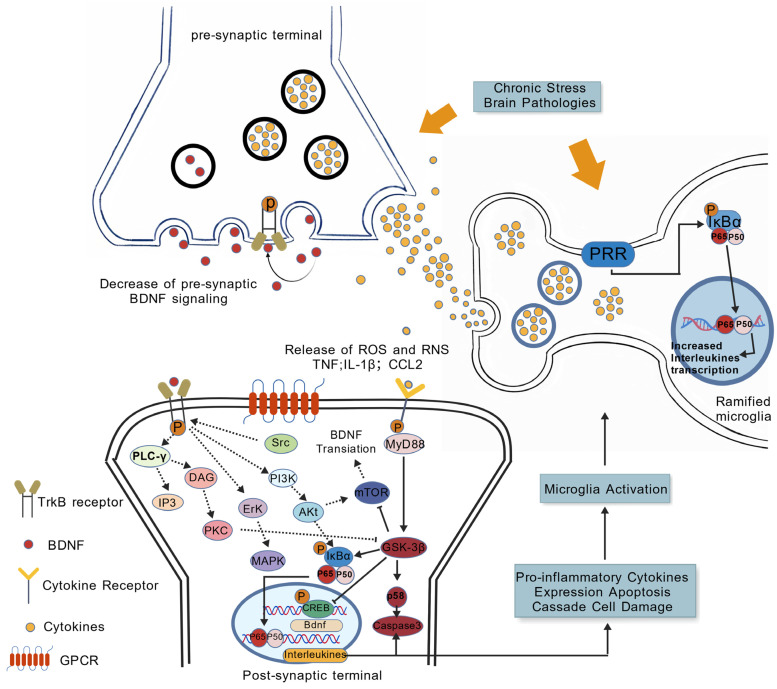
Schematic summary of the NF-κB dependent proinflammatory pathway. Created with BioGDP.com [50].

Inflammation is a protective mechanism in the human body that automatically defends against external foreign bodies or self-generated foreign bodies. It is a complex protective response to biological, chemical, and physical injuries, caused by signaling molecules produced by white blood cells, macrophages, and mast cells, as well as the activation of complement factors. These complement factors extravasate at the site of inflammation, leading to edema [61]. Inflammation can induce changes in multiple intracellular signal cascades, ultimately leading to severe consequences such as neurological dysfunction, cell apoptosis, cognitive impairment, behavioral abnormalities, and neurological and psychiatric disorders (Figure 3) [62,63]. Fingered citron volatile oil also exhibits good anti-inflammatory properties. Studies by Sun et al. [64] have shown that fingered citron volatile oil can reduce the levels of pro-inflammatory factors TNF-α, IL-1α, and IL-6 in the serum of golden hamsters, increase the activity of Caspase-3 in sebaceous gland cells, inhibit the growth rate of sebaceous glands and the accumulation of triglycerides, thereby showing anti-inflammatory effects. Kim et al. [65] found that α-pinene in fingered citron volatile oil can inhibit inflammation stimulated by bacterial cell wall lipopolysaccharide (LPS) by suppressing mitogen-activated protein kinases and the NF-κB pathway in mouse peritoneal macrophages, thereby exhibiting anti-inflammatory activity. Lombardo et al. [66] demonstrated that intraperitoneal injection of fingered citron volatile oil fractions with furocoumarins removed can inhibit carrageenan-induced paw edema in rats by reducing the release of pro-inflammatory cytokines such as interleukin (IL)-1β, IL-6, and tumor necrosis factor (TNF)-α.

### 5.7. Improving Skin Vitiligo

Vitiligo is an autoimmune skin disease caused by melanocyte dysfunction, clinically characterized by depigmented skin patches. The onset of vitiligo is related to factors such as genetics, oxidative stress, inflammation, and environmental triggers [67]. 5-methoxypsoralen, also known as bergapten, is a naturally occurring furocoumarin, which, when used in combination with ultraviolet A (UVA), has therapeutic effects on skin diseases such as psoriasis, vitiligo, and atopic eczema. Shaaban et al. [68] demonstrated that fingered citron volatile oil is a photosensitizer that can be used in photodynamic therapy (PDT) for the treatment of skin diseases such as vitiligo. It can be integrated into the lipid matrix of nanostructured lipid carriers (NLCs) as a liquid lipid component, exerting dual effects: improving photostability and photodynamic characteristics, enhancing the efficiency and safety of photodynamic therapy using fingered citron volatile oil for vitiligo, and strengthening the pigment repigmentation response in vitiligo patients. It has shown promising results in preclinical and clinical studies of topical photodynamic therapy for vitiligo.

### 5.8. Antioxidation

Fingered citron volatile oil has antioxidant activity. Huang Weichao et al. [69] conducted in vitro antioxidant experiments on fingered citron volatile oil, which also showed that fingered citron volatile oil has a high scavenging rate of DPPH, ABTS, and hydroxyl free radicals. Xing et al. [70] determined the scavenging effect of fingered citron volatile oil on superoxide anion free radicals (O_2_^−^·). The scavenging rate of fingered citron volatile oil for O_2_^−^· showed a dose-dependent manner. When the mass concentration of fingered citron volatile oil was 10 mg/mL, the scavenging rate was only slightly lower than that of vitamin C, indicating that fingered citron volatile oil has strong antioxidant activity. At present, there are many studies on the antioxidant effect of plant essential oils, but most of them are limited to the antioxidant efficiency of essential oils, while studies on the mechanism of antioxidant activity of plant essential oils are very rare. In the future, in-depth research can be carried out on the mechanism of antioxidant activity of essential oils.

### 5.9. Pain Relief

Fingered citron volatile oil can alter the plasticity of normal and pathological protrusions, which is related to nociceptive and neuropathic pain. Studies have shown that fingered citron volatile oil regulates the sensitive perception of pain in different types of nociceptive, inflammatory, and neuropathic pain [71]. Scuteri et al. [72] demonstrated that inhalation of fingered citron volatile oil can reduce the licking and biting behavior in mice with nociceptive pain induced by plantar injection of formalin, suggesting that fingered citron volatile oil has the effect of relieving pain. Another study showed that subcutaneous injection of fingered citron volatile oil or linalool into the plantar of the ipsilateral hind paw of mice can reduce the formalin-induced licking and biting response in mice, and the effect of linalool is better than that of fingered citron volatile oil, indicating that both fingered citron volatile oil and linalool have the effect of relieving pain [73].

### 5.10. Antibacterial Effect

An increase in pathogenic bacteria and a decrease in beneficial bacteria can lead to intestinal flora imbalance, resulting in gastroenteritis. In addition, pathogenic bacteria can also cause foodborne diseases, respiratory infections, and even death. Natural active substances are less likely to induce drug resistance, providing a new strategy to combat pathogenic bacteria resistance. Fingered citron volatile oil exhibits good antibacterial activity against a variety of bacteria and fungi. Studies have shown that limonene contained in fingered citron has an inhibitory effect on Diplococcus hepatitis and Staphylococcus aureus. LI et al. [40] found that fingered citron volatile oil exhibits moderate antibacterial activity against common foodborne bacteria such as Escherichia coli, Staphylococcus aureus, Bacillus subtilis, and Micrococcus flavus. By observing changes in bacterial morphology through scanning electron microscopy, time-kill analysis, cell permeability, and membrane integrity assays, it was found that fingered citron volatile oil significantly inhibits the growth rate of viable bacteria. Its antibacterial mechanism may be that it causes cell wall dissolution and leakage of intracellular components, thereby leading to cell death.

## 6. Development and Problems of Fingered Citron Volatile Oil Products

### 6.1. Development of Fingered Citron Volatile Oil Products

In view of the various pharmacological effects of different components in fingered citron, the development of various fingered citron-related products has increasingly become a research hotspot at present, and related products with fingered citron as the main raw material have been successively launched. As a precious spice, fingered citron has broad market prospects, and its rational development and comprehensive utilization will generate considerable economic and social benefits. Abroad, fingered citron volatile oil is regarded as an elegant fragrance and applied to various foods and cosmetics, with in-depth and detailed research conducted on its medicinal value and other aspects. Compared with some Western developed countries, the application of fingered citron volatile oil in China is just in its infancy, with initial applications in various industries such as food, medicine, cosmetics, and daily necessities. For example, it is used in aromatherapy, as air fresheners, perfumes, beverages, chewing gum, condiments, etc. With the continuous deepening of research on fingered citron volatile oil and the increasing maturity of related extraction and application technologies, this natural product with extensive potential uses will be gradually promoted and applied.

#### 6.1.1. Fingered Citron Volatile Oil Can Be Used to Develop High-Aroma and Low-Tar Cigarette Products

Studies have found that essential oils play a special role in the production of low-tar tobacco and can be used to develop high-aroma and low-tar cigarette products. In the production of low-tar cigarettes, much of the tobacco aroma is lost. However, adding aroma-producing substances like essential oils to low-tar cigarettes can make up for the loss of flavor substances during production, thereby enabling the development of high-aroma and low-tar cigarette products. Although the application of fingered citron in cigarettes has not yet been studied, fingered citron has a high content of essential oil. If further analysis and research can be conducted on fingered citron volatile oil and it can be applied to cigarettes, it will have significant practical significance and broad application prospects.

#### 6.1.2. Fingered Citron Volatile Oil Microcapsules

It has been reported in studies that fingered citron volatile oil can be prepared into fingered citron volatile oil microcapsules using spray drying technology, with β-cyclodextrin and gum arabic as wall materials and fingered citron volatile oil as the raw material, through single-factor experiments and response surface methodology. The optimal process parameters for fingered citron volatile oil microcapsules were optimized in the experiment, and the embedding rate of the resulting product was 10.65%. These microcapsules exhibit functions such as sustained release, controlled release, and improved targeting, which are of great significance for expanding the application scope and enhancing the application capability of fingered citron volatile oil [40].

#### 6.1.3. Fingered Citron Volatile Oil Insecticides

In recent years, with the continuous deepening of relevant research, people have gradually found that chemical insecticides have adverse effects such as great harm to the human body and the development of pest resistance. In recent years, the exploration of plant-derived insecticides has gradually emerged [74]. Essential oil substances usually have the synergistic effect of multiple components, and their insecticidal mechanisms and targets are complex, making it difficult for pests to develop resistance. At the same time, plant essential oils are usually highly selective and relatively safe for humans and animals in household environments. Fingered citron volatile oil has certain acaricidal activity, which may be related to the D-limonene component contained in fingered citron volatile oil. Several literature sources have confirmed that fingered citron volatile oil has certain acaricidal activity, but there are problems such as large dosage and high price when used alone. To expand its utilization, the compound combination of fingered citron volatile oil and essential oils reported to have insecticidal effects on the market can be explored to reduce the cost of subsequent product development and improve the utilization of essential oil products [75].

#### 6.1.4. Application of Fingered Citron Volatile Oil in Fruit and Vegetable Preservation

Studies on the preservation effect of natural plant extracts on fruits and vegetables have been reported, among which plant essential oils have shown good effects on the post-harvest preservation of various fruits and vegetables such as strawberries, apples, and peppers. As a natural plant extract, fingered citron volatile oil is considered to have a good application prospect in the post-harvest preservation of agricultural products due to its characteristics of volatility, strong activity, and antiseptic and antibacterial effects. Studies have proved that fingered citron volatile oil can well maintain the appearance quality, soluble solid content, reducing sugar content, and protective enzyme activity of post-harvest plum fruits, and can effectively inhibit the respiration rate and membrane lipid peroxidation during storage, showing a good preservation effect. This provides a certain theoretical basis for the application and development of essential oils in fruit and vegetable preservation.

#### 6.1.5. Fingered Citron Volatile Oil Gummies

Plant essential oils are often used to extend the shelf life of food due to their antioxidant and antibacterial activities and can also be used to improve the taste and flavor of food. Traditional Chinese medicine functional gummies have changed the unpleasant taste and inadaptability of traditional dosage forms. They have health-care effects such as enhancing immunity and assisting in lowering blood sugar and blood lipids, which cater to consumers’ physical and psychological needs, thus attracting widespread attention [76]. As a type of plant essential oil, fingered citron volatile oil has been proven to have various high activities such as antioxidant, antibacterial, anti-inflammatory, and analgesic effects, so it is also suitable for the food field. Tang Niang first developed a kind of fingered citron volatile oil gummy with fingered citron aroma and certain functional properties using fingered citron volatile oil as a raw material and studied the optimal formula of fingered citron volatile oil gummies. The final gummies have moderate hardness, delicate taste, bright color, moderate sourness and sweetness, softness with elasticity, and possess the aroma of fingered citron volatile oil [77]. The application of fingered citron volatile oil in food is of great significance for the development of the intensive processing industry of fingered citron and the application of fingered citron volatile oil in the field of functional food.

### 6.2. Problems Faced in the Development of Fingered Citron Volatile Oil Products

However, it should be noted that fingered citron volatile oil may have potential phototoxicity. Many citrus essential oils may contain furanocoumarins, and these compounds are closely associated with phototoxic reactions after exposure to ultraviolet light or sunlight. If essential oil containing photosensitive components is directly applied to sensitive skin and subsequently exposed to strong light, ultraviolet radiation, or sunlight, it may induce photosensitive contact dermatitis, pigmentation, or other adverse skin reactions [12]. Therefore, reducing or removing furanocoumarins is an important strategy for improving the safety of fingered citron volatile oil products. Chen et al. [78] determined the contents of furanocoumarins in four commercially available citrus essential oils used for aromatherapy and performed defuranocoumarination experiments using liquid–liquid extraction, which contributed to the development of safer desensitized essential oils. In addition, Chen et al. [4] reported that bergapten could not be extracted from fingered citron using steam distillation or hydrodistillation. These findings suggest that extraction method optimization and furanocoumarin removal are important approaches for reducing phototoxic risk. In future product development, safety evaluation, concentration control, appropriate labeling, and avoidance of ultraviolet exposure after topical application should also be considered.

## 7. Results and Discussion

Fingered citron volatile oil has good development prospects in fields such as pharmaceuticals and cosmetics, with extremely high research value. This review summarized and analyzed eight extraction methods of volatile oils and concluded that the ultrasonic-assisted steam extraction method not only retains the advantages of the steam distillation method but also shortens the extraction time and improves extraction efficiency, making it a better method for extracting fingered citron volatile oil. This paper sorted out 90 components of fingered citron volatile oil. Studying the chemical components of fingered citron volatile oil can not only help research its biological activities but also promote the development of natural fragrances.

In terms of research on biological activities, available studies on fingered citron volatile oil, together with supportive evidence from related citrus essential oils where direct evidence is limited, indicate potential activities such as relieving anxiety, antidepressant activity, anti-tumor activity, anti-inflammatory activity, antioxidant activity, antibacterial activity, relieving cough and asthma, improving insomnia, and benefiting skin conditions such as vitiligo, acne, and osteoporosis. Traditional Chinese medicine theory holds that fingered citron has the function of soothing the liver and regulating qi and can relieve cough and asthma. However, there are few reports at home and abroad on the effects of fingered citron volatile oil on cough and asthma, and the existing research is mainly limited to chemical composition analysis and preliminary efficacy observation. In future studies, further pharmacological validation and mechanistic research are needed to clarify its specific active components and biological effects.

At present, as a plant with both medicinal and edible values, fingered citron and its volatile oil are mostly used in food and cosmetics. The food products include various fingered citron-based products such as dried fruits, fingered citron honey extract, fingered citron tea, and fingered citron vinegar. In the future, China can develop targeted ordinary fingered citron foods and functional foods based on the biological activities of fingered citron volatile oil. This will not only meet people’s demand for healthy foods but also enable the application of fingered citron volatile oil in the production of various food products. Fingered citron volatile oil may have potential value in emotional regulation. Applying it in cosmetics can strengthen research on its benefits for skin and human health, with attention to its possible anxiety- and depression-related effects. Moreover, fingered citron volatile oil can be combined with other essential oils or Chinese medicinal materials to establish multiple fingered citron industrial chains, promote its development, and gradually popularize the application of this natural product with extensive potential uses.

## Figures and Tables

**Table 1:** Comparison of advantages and disadvantages of different extraction methods for fingered citron volatile oil.

Extraction Method	Heat Addition	Advantages	Disadvantages	Optimal Process	Applicable Scenarios	References
Cold Pressing Method	×	High quality essential oil	Immature technology	Processing in an environment below 60 °C, with the most complete retention of nutrients	Industrial large-scale continuous production	[13]
SD Method	√	Simple process, low cost, stable yield, no organic residue, good quality volatile oil without impurities	Low extraction efficiency; essential oil components are destroyed and easily hydrolyzed to form useless impurities	No NaCl added, solid–liquid ratio for distillation is 1:14, distillation time is 7 h	Laboratory and small-scale production	[14]
MD Method	√	Low pressure, short time, low operating temperature, high separation degree, good product quality	Complex equipment structure, high energy consumption, low production efficiency, high cost	Distillation temperature 35 °C, vacuum degree 200 Pa, scraper rotation speed 250 r/min, feeding speed 0.3 L/h, target component content increased from 44.2% to 75.3%	Laboratory and small-scale production	[15]
Solvent Extraction Method	×	Simple operation, wide applicability, high extraction efficiency, mild conditions, less component damage, low cost	High time cost, environmental pollution, solvent residue	Using petroleum ether as extraction solvent, solid–liquid ratio 24:1, reflux extraction twice, 60 min each time	Separation of specific components	[16]
SFE Method	√	Simple process, few steps, high yield, green and environmentally friendly, simple technology	Large equipment investment, high technical requirements for the process	Extraction pressure 40 MPa, extraction temperature 60 °C, separation temperature 35 °C, separation pressure 10 MPa	Industrial production, high-value-added products	[17]
Ultrasonic-Assisted Extraction Method	×	High extraction efficiency, short extraction time, simple operation, wide application range, small solvent dosage	Large initial investment, high equipment cost, limited component selectivity	Water distillation flow rate 1.30 mL/min, ultrasonic power 180 W, auxiliary working time 30 min, water vapor distillation time 75 min, the extraction rate of fingered citron volatile oil is 0.763%	Laboratory or small-scale production	[18]
SDE Method	√	Simultaneously realizes separation, concentration and solvent conversion	Long operation time, complex device		Laboratory and small-to-medium-scale production	[19]
Enzyme-Assisted Extraction Method	√	Maintains the activity and stability of target components; simple operation, environmentally friendly and non-toxic	High cost, strict reaction conditions, long time consumption		Laboratory or small-scale production	[20]

“√” indicates that heating was applied, whereas “×” indicates that no heating was applied.

**Table 2:** The main chemical components in fingered citron volatile oil.

Number	Name	Type	Structural Formula	Molecular Formula	Molecular Weight	References
1	3-hydroxy-2-butanone	alcohols	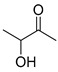	C_4_H_8_O_2_	88.11	[20]
2	2,3-butanediol	alcohols	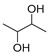	C_4_H_10_O_2_	90.12	[20]
3	methylthiopropanal	sulfur compounds	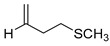	C_4_H_8_O_S_	104.17	[20]
4	valeraldehyde	aldehydes	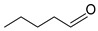	C_5_H_10_O	86.13	[20]
5	2-ethoxybutane	ethers	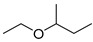	C_6_H_14_O	102.17	[20]
6	Nonyl aldehyde	aldehydes	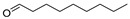	C_9_H_18_O	142.24	[20]
7	ocimene	monoterpenes	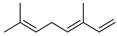	C_10_H_16_	136.23	[20]
8	2-Undecanone	ketones	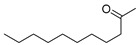	C_11_H_22_O	170.29	[20]
9	2-Tridecanone	ketones	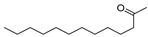	C_13_H_26_O	198.34	[20]
10	Geranyl isobutyrate	esters	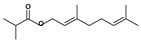	C_14_H_24_O_2_	224.34	[20]
11	Methyl myristate	esters	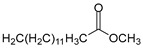	C_15_H_30_O_2_	242.4	[20]
12	methyl hexadecanoate	esters	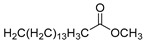	C_17_H_34_O_2_	270.45	[20]
13	methyl linoleate	esters	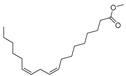	C_19_H_34_O_2_	294.47	[20]
14	ethyl linoleate	esters	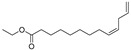	C_20_H_36_O_2_	308.5	[20]
15	2-Isopropyltoluene	monoterpenes	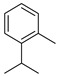	C_10_H_14_	134.22	[20]
16	p-cymene	monoterpenes	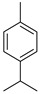	C_10_H_14_	134.22	[30]
17	m-cymene	monoterpenes	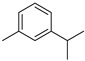	C_10_H_14_	134.22	[20]
18	α-Terpinene	monoterpenes	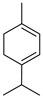	C_10_H_16_	136.23	[30]
19	terpinolene	monoterpenes	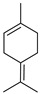	C_10_H_16_	136.23	[30]
20	α-phellandrene	monoterpenes	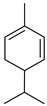	C_10_H_16_	136.23	[30]
21	β-phellandrene	monoterpenes	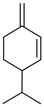	C_10_H_16_	136.23	[10]
22	γ-terpinene	monoterpenes	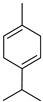	C_10_H_16_	136.23	[31]
23	limonene	monoterpenes	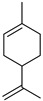	C_10_H_16_	136.23	[32]
24	Phenylacetaldehyde	aldehydes	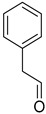	C_8_H_8_O	120.15	[20]
25	Anethole	phenolic ethers	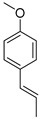	C_10_H_12_O	148.20	[20]
26	4-isopropyl-3-methylphenol	phenols	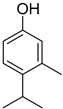	C_10_H_14_O	150.22	[20]
27	Thymol	phenols	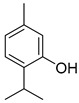	C_10_H_14_O	150.22	[20]
28	Eugenol	phenols	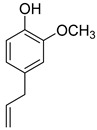	C_10_H_12_O_2_	164.20	[20]
29	perillyl alcohol	alcohol	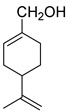	C_10_H_16_O	152.23	[20]
30	(-)-Carveol	alcohol	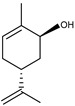	C_10_H_16_O	152.23	[33]
31	cis-carveol	alcohol	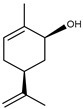	C_10_H_16_O	152.23	[33]
32	Piperitone	ketones	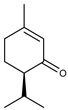	C_10_H_16_O	152.23	[34]
33	Terpinen-4-ol	alcohol	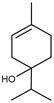	C_10_H_18_O	154.25	[35]
34	Isopulegol	alcohol	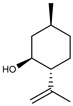	C_10_H_18_O	154.25	[34]
35	1,8-cineole	ethers	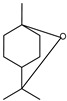	C_10_H_18_O	154.25	[36]
36	Acetic acid ester parsley	esters	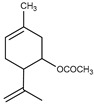	C_12_H_18_O_2_	194.27	[37]
37	Perillyl acetate	esters	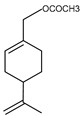	C_12_H_18_O_2_	194.27	[36]
38	α-terpinyl acetate	esters	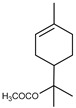	C_12_H_20_O_2_	196.29	[36]
39	β-myrcene	monoterpenes	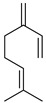	C_10_H_16_	136.23	[35]
40	Ocimene	monoterpenes	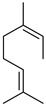	C_10_H_16_	136.24	[37]
41	β-citral	aldehydes	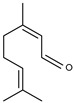	C_10_H_16_O	152.23	[38]
42	α-citral	aldehydes	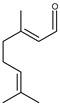	C_10_H_16_O	152.23	[32]
43	Nerol	alcohol	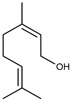	C_10_H_18_O	154.25	[37]
44	Citronellal	aldehydes	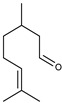	C_10_H_18_O	154.25	[33]
45	Geraniol	alcohol	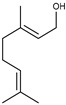	C_10_H_18_O	154.25	[33]
46	β-linalool	alcohol	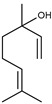	C_10_H_18_O	154.25	[33]
47	Geranyl acetate	esters	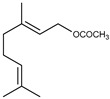	C_12_H_20_O_2_	196.29	[33]
48	Neryl acetate	esters	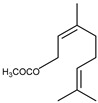	C_12_H_20_O_2_	196.29	[33]
49	Citronellyl acetate	esters	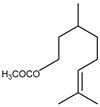	C_12_H_22_O_2_	198.30	[33]
50	Linalyl acetate	esters	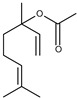	C_12_H_20_O_2_	196.29	[37]
51	α-pinene	monoterpenes	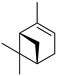	C_10_H_16_	136.23	[35]
52	β-pinene	monoterpenes	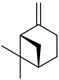	C_10_H_16_	136.23	[32]
53	Verbenone	ketones	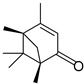	C_10_H_14_O	150.22	[33]
54	Thujene	monoterpenes	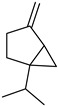	C_10_H_16_	136.23	[39]
55	4-carene	monoterpenes	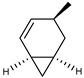	C_10_H_16_	136.23	[33]
56	3-carene	monoterpenes	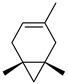	C_10_H_16_	136.23	[40]
57	camphene	monoterpenes	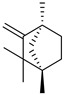	C_10_H_16_	136.23	[37]
58	tricyclene	monoterpenes	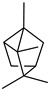	C_10_H_16_	136.23	[34]
59	beta-cyclocitral	aldehydes	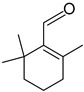	C_10_H_16_O	152.23	[20]
60	Linalool oxide	ethers	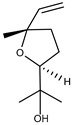	C_10_H_18_O_2_	170.25	[34]
61	β-elemene	sesquiterpenes	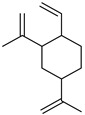	C_15_H_24_	204.35	[39]
62	γ-elemene	sesquiterpenes	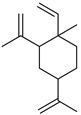	C_15_H_24_	204.35	[39]
63	δ-elemene	sesquiterpenes	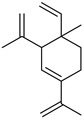	C_15_H_24_	204.35	[39]
64	γ-cadinene	sesquiterpenes	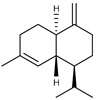	C_15_H_24_	204.35	[37]
65	δ-cadinene	sesquiterpenes	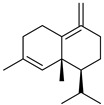	C_15_H_24_	204.35	[35]
66	γ-muurolene	sesquiterpenes	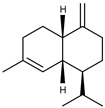	C_15_H_24_	204.35	[37]
67	germacrene D	sesquiterpenes	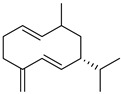	C_15_H_24_	204.35	[33]
68	germacrene B	sesquiterpenes	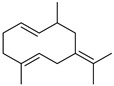	C_15_H_24_	204.35	[33]
69	germacrene	sesquiterpenes	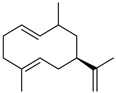	C_15_H_24_	204.35	[35]
70	bicyclogermacrene	sesquiterpenes	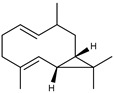	C_15_H_24_	204.35	[39]
71	α-bisabolol	alcohol	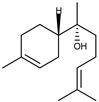	C_15_H_26_O	222.37	[35]
72	trans-α-bisabolene	sesquiterpenes	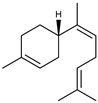	C_15_H_24_	204.35	[35]
73	β-bisabolene	sesquiterpenes	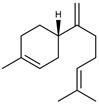	C_15_H_24_	204.35	[33]
74	β-caryophyllene	sesquiterpenes	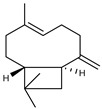	C_15_H_24_	204.35	[37]
75	isocaryophyllene	sesquiterpenes	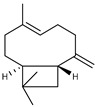	C_15_H_24_	204.35	[33]
76	caryophyllene oxide	sesquiterpenes	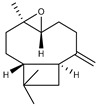	C_15_H_24_O	220.35	[35]
77	spathulenol	sesquiterpenes	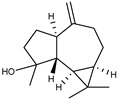	C_15_H_24_O	220.35	[41]
78	β-patchoulene	sesquiterpenes	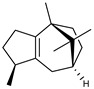	C_15_H_24_	204.35	[35]
79	β-gurjunene	sesquiterpenes	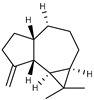	C_15_H_24_	204.35	[37]
80	Patchouli alcohol	alcohol	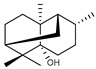	C_15_H_26_O	222.37	[35]
81	α-caryophyllene	sesquiterpenes	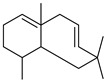	C_15_H_24_	204.35	[35]
82	β-santalene	sesquiterpenes	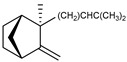	C_15_H_24_	204.35	[37]
83	α-cadinene	sesquiterpenes	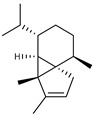	C_15_H_24_	204.35	[35]
84	β-cadinene	sesquiterpenes	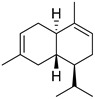	C_15_H_24_	204.35	[39]
85	campherenol	alcohol	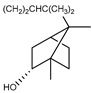	C_15_H_26_O	222.37	[34]
86	α-cis-bergamotene	sesquiterpenes	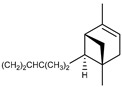	C_15_H_24_	204.35	[39]
87	copaene	sesquiterpenes	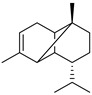	C_15_H_24_	204.35	[37]
88	trans-β-farnesene	sesquiterpenes	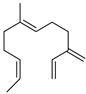	C_15_H_24_	204.35	[35]
89	α-Ionone	ketones	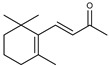	C_13_H_20_O	192.30	[39]
90	2,2′-Methylenebis- (6-tert-butyl-4-methylphenol)	phenols	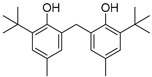	C_23_H_32_O_2_	340.50	[20]

## Data Availability

No new data were generated or analyzed during the preparation of this review. Data sharing is not applicable to this article.

## Abbreviations

The following abbreviations are used in this manuscript:

SD	Steam distillation
GC-MS	gas chromatography-mass spectrometry
HPLC	high-performance liquid chromatography
NMR	nuclear magnetic resonance spectroscopy
MD	molecular distillation
SE	solvent extraction
SFE	supercritical fluid extraction
SDE	simultaneous distillation-extraction
LPS	lipopolysaccharide
UVA	ultraviolet A
PDT	photodynamic therapy
NLCs	nanostructured lipid carriers
5-HT	5-hydroxytryptamine
HPLC-IT-TOF-MS	high-performance liquid chromatography-ion trap time-of-flight mass spectrometry
